# Supplementary material for: Large-scale automated detection reveals pervasive sex imbalance in biomedical research
Source: bioRxiv. 2026 Jul 14:2026.07.13.738332. Preprint. [Version 1] doi: 10.64898/2026.07.13.738332 (PMC13404891; doi:10.64898/2026.07.13.738332)
Supplement: Supplement 1 [file media-1.pdf]

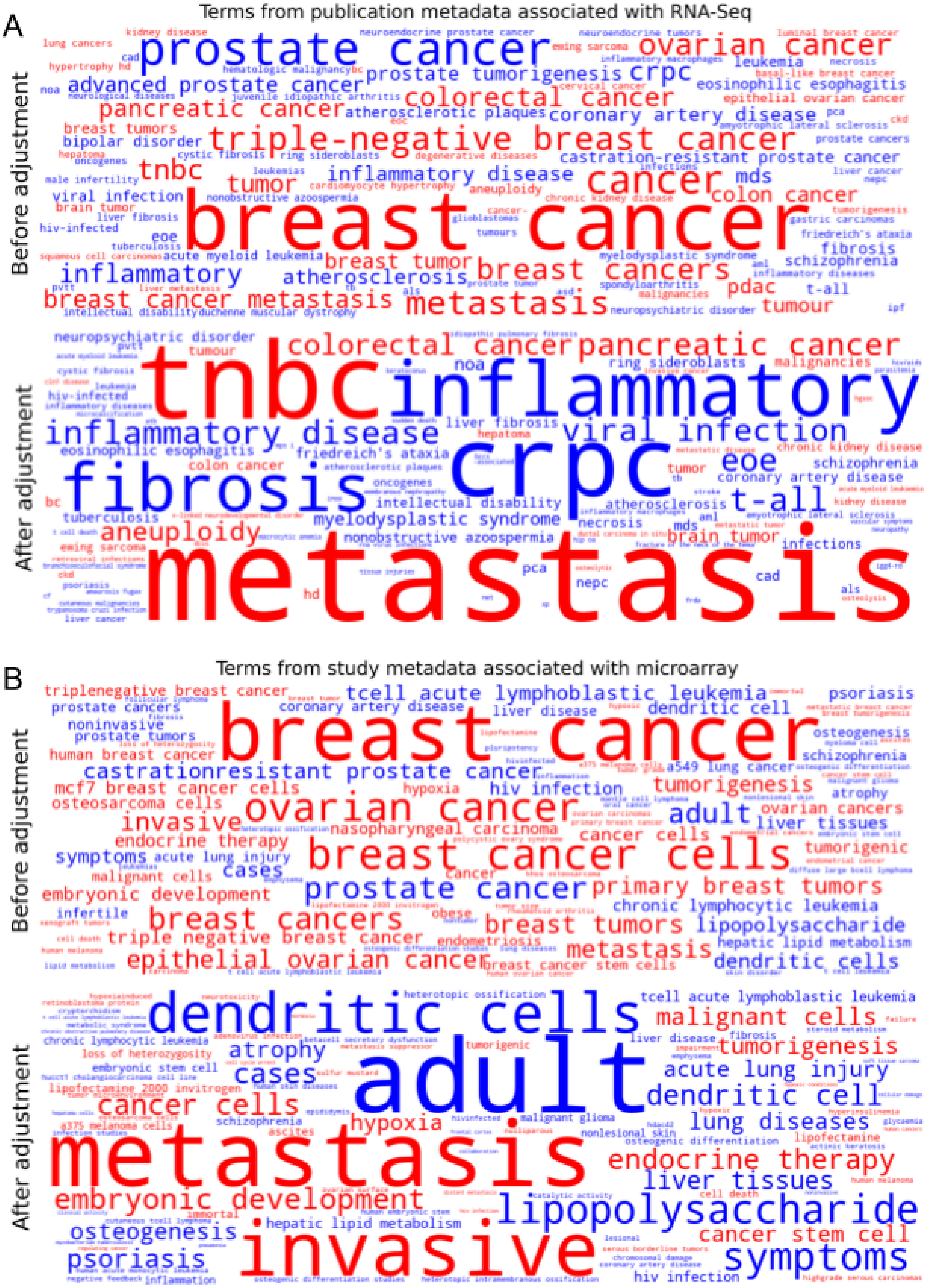
A
Terms from publication metadata associated with RNA-Seq
Before adjustment
breast cancer
prostate cancer
ovarian cancer
triple-negative breast cancer
colorectal cancer
pancreatic cancer
advanced prostate cancer
prostate tumorigenesis
crpc
cancer
tnbc
tumor
inflammatory disease
inflammatory
breast cancers
breast cancer metastasis
metastasis
atherosclerosis
breast tumor
coronary artery disease
castration-resistant prostate cancer
colon cancer
mds
pdac
tumour
leukemia
eosinophilic esophagitis
schizophrenia
fibrosis
t-all
viral infection
bipolar disorder
breast tumors
acute myeloid leukemia
After adjustment
metastasis
tnbc
inflammatory
crpc
fibrosis
inflammatory disease
viral infection
colorectal cancer
pancreatic cancer
aneuploidy
eoe
t-all
myelodysplastic syndrome
eosinophilic esophagitis
friedreich's ataxia
schizophrenia
coronary artery disease
atherosclerosis
intellectual disability
nonobstructive azoospermia
brain tumor
B
Terms from study metadata associated with microarray
Before adjustment
breast cancer
breast cancer cells
ovarian cancer
prostate cancer
breast cancers
breast tumors
epithelial ovarian cancer
tcell acute lymphoblastic leukemia
castrationresistant prostate cancer
primary breast tumors
lipopolysaccharide
metastasis
dendritic cells
dendritic cell
tumorigenesis
adult
invasive
endocrine therapy
triple negative breast cancer
triplenegative breast cancer
mcf7 breast cancer cells
osteosarcoma cells
embryonic development
chronic lymphocytic leukemia
nasopharyngeal carcinoma
psoriasis
osteogenesis
schizophrenia
After adjustment
adult
metastasis
invasive
dendritic cells
dendritic cell
lipopolysaccharide
symptoms
embryonic development
endocrine therapy
malignant cells
tumorigenesis
acute lung injury
lung diseases
liver tissues
cancer cells
atrophy
cases
hypoxia
osteogenesis
psoriasis
cancer stem cell
hepatic lipid metabolism
tcell acute lymphoblastic leukemia

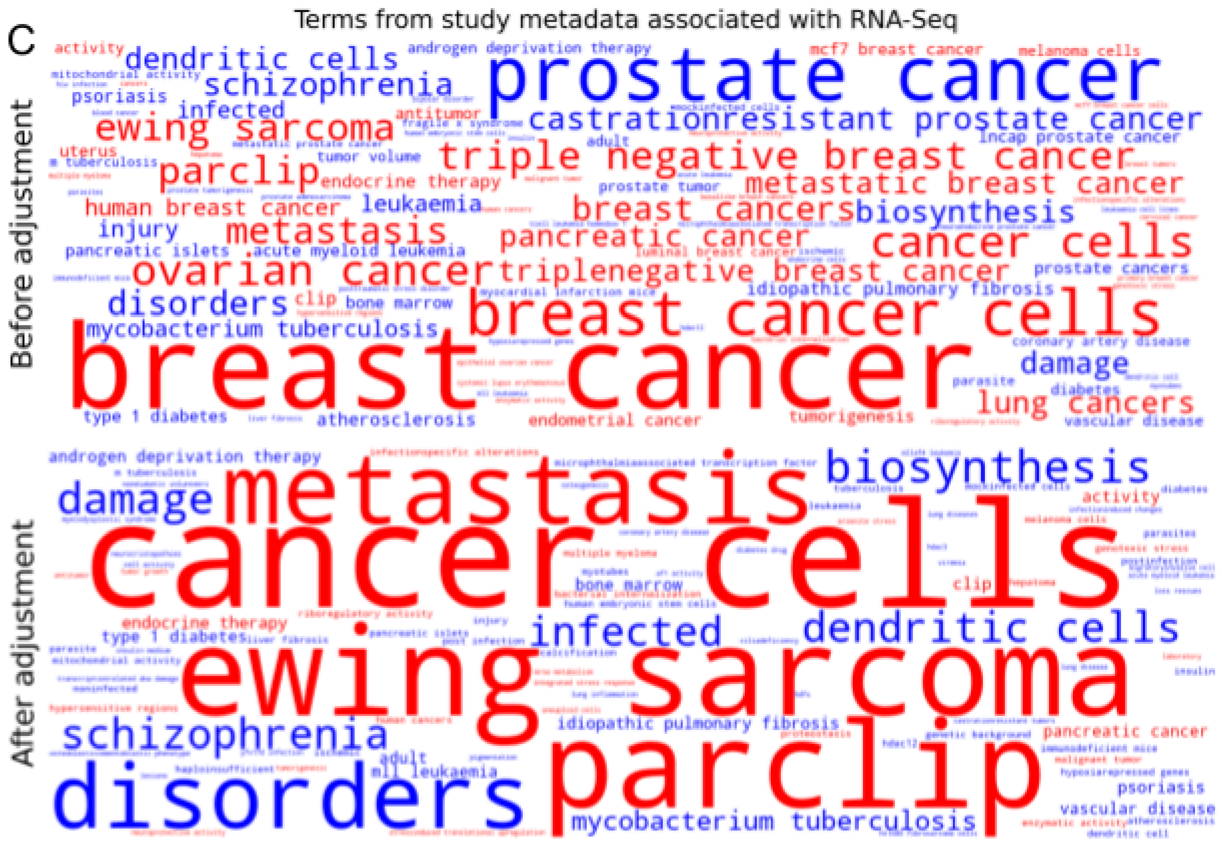

Supplemental Figure 1. Terms from titles and abstracts of publications associated with RNA-Seq (A) samples and from descriptions of studies associated with microarray (B) and RNA-Seq (C) samples. Red word color indicates terms skewed toward female representation and blue word color indicates terms skewed toward male representation.
